# The Nursing Mirror

**Published:** 1888-02-04

**Authors:** 


### THE NURSING MIRROR.

Communications for this column should be addressed The Mirror, care of the Editor, 38, Old Jewry, London, E.C.

### EXAMINATIONS FOR CERTIFICATES.

I SHOULD like to make one or two suggestions with regard to the question of competitive examinations for nurses.

As to the "modern craze" for certificates, as "Kittifonia" puts it, at any rate there is method in our madness, and seeing that the holding certificates has become so important a matter in almost every profession, we must endeavour to swim with the stream.

I should be glad to see the question pushed forward in THE HOSPITAL, and put into practical shape by The Hospitals Association, for at present we provincial trained nurses have not a fair chance with those trained in a London school, though the training may be equally good.

If all nurses were to compete for a general examination in connexion with The Hospitals Association, it would give us more equal chances all round, and I do not see that "Kittifonia's" "excellent nurses," who apparently have such a horror of examinations, need be a barrier to the scheme.

I would suggest—

1st. That the examinations be held twice in the year, and that each training school form a centre.

2nd. That the papers of questions be issued by The Hospitals Association only.

3rd. That candidates may pass first, second, and third class, those being allowed who have passed only third class to enrol themselves as candidates a second time if they so desire.

4th. That those who are trained in very small hospitals be allowed to attend at the nearest centre for examination, which would obviate in a great measure the pecuniary difficulty.

5th. That no certificate be considered complete unless it be supplemented by a written statement from the matron or medical officer under whom the nurse has been trained, certifying her competency in the practical duties of nursing, and also her good character and conduct.

I submit the above suggestions to your consideration, and wish "M.B.'s" scheme all success.

January 28th, 1888. ALICE.

